# The Expression Pattern of the Na^+^ Sensor, Na_X_ in the Hydromineral Homeostatic Network: A Comparative Study between the Rat and Mouse

**DOI:** 10.3389/fnana.2012.00026

**Published:** 2012-07-19

**Authors:** Benjamin Nehmé, Mélaine Henry, Didier Mouginot, Guy Drolet

**Affiliations:** ^1^Axe Neurosciences du CRCHUQ (CHUL), Faculté de Médecine, Université LavalQuébec, QC, Canada

**Keywords:** sodium channel, *Scn7a*, magnocellular neurons, circumventricular organs, sodium homeostasis, Na_X_ channel

## Abstract

The Scn7a gene encodes for the specific sodium channel Na_X_, which is considered a primary determinant of sodium sensing in the brain. Only partial data exist describing the Na_X_ distribution pattern and the cell types that express Na_X_ in both the rat and mouse brain. To generate a global view of the sodium detection mechanisms in the two rodent brains, we combined Na_X_ immunofluorescence with fluorescent cell markers to map and identify the Na_X_-expressing cell populations throughout the network involved in hydromineral homeostasis. Here, we designed an anti-Na_X_ antibody targeting the interdomain 2–3 region of the Na_X_ channel’s α-subunit. In both the rat and mouse, Na_X_ immunostaining was colocalized with vimentin positive cells in the median eminence and with magnocellular neurons immunopositive for neurophysin associated with oxytocin or vasopressin in both the supraoptic and paraventricular nuclei. Na_X_ immunostaining was also detected in neurons of the area postrema. In addition to this common Na_X_ expression pattern, several differences in Na_X_ immunostaining for certain structures and cell types were found between the rat and mouse. Na_X_ was present in both NeuN and vimentin positive cells in the subfornical organ and the vascular organ of the lamina terminalis of the rat whereas Na_X_ was only colocalized with vimentin positive cells in the mouse circumventricular organs. In addition, Na_X_ immunostaining was specifically observed in NeuN immunopositive cells in the median preoptic nucleus of the rat. Overall, this study characterized the Na_X_-expressing cell types in the network controlling hydromineral homeostasis of the rat and mouse. Na_X_ expression pattern was clearly different in the nuclei of the lamina terminalis of the rat and mouse, indicating that the mechanisms involved in systemic and central Na^+^ sensing are specific to each rodent species.

## Introduction

The sodium (Na^+^) channel Na_X_ is atypical in the type 1 voltage-gated Na^+^ channel family (Na_v_1.1–Na_v_1.9), regarding its α-subunit sequence. Indeed, the Na_X_ channel shares <50% sequence identity with this Na^+^ channel family and the structural specificity of the Na_X_ channel lies in the marked discrepancies within the consensus amino acid sequences characterizing the voltage sensor domain (S4 segment), the pore region (P domain), and the inactivation gate (interdomain region 3; Goldin et al., 2000). Presently, there is a consensus regarding the grouping of the Na_X_ channel (gene symbol *Scn7a*) with several other atypical Na^+^ channels that are strongly homologous to each other (>80% overall identity). These Na^+^ channels were previously cloned (fully or partially) from cultured astrocytes (NaG; Gautron et al., 1992), human heart and uterus (Na_v_2.1; George et al., 1992), atrial tumor cell line (Na_v_2.3; Felipe et al., 1994), and dorsal root ganglion (*Scl11*; Akopian et al., 1997). Therefore, Na_v_2.3, NaG or *Scl11* refer to previous nomenclature of the Na_X_ channel (Goldin et al., 2000; Watanabe et al., 2000).

The local expression and functional role of the Na_X_ channel in the brain indicate that Na_X_ is a critical determinant of Na^+^ homeostasis. In animal models, the creation of a Na_X_ deficient mouse in which the *lacZ* gene was inserted into the first exon of the Na_V_2.3 gene (such that it was expressed as a fusion protein with β-galactosidase) provided the first indication of both the brain localization and the functional properties of this atypical Na^+^ channel *in vivo* (Watanabe et al., 2000). An analysis of the β-galactosidase activity in the heterozygous mutant mice (Na_v_2.3^±^) demonstrated a restricted pattern of Na_v_2.3 (Na_X_) throughout the central nervous system with its highest expression in the four following circumventricular organs (CVOs): the subfornical organ (SFO), the vascular organ of the lamina terminalis (OVLT), the median eminence (ME), and the neurohypophysis. The presence of Na_V_2.3 in brain regions that are critical for hydromineral balance is in agreement with the putative function of this atypical Na^+^ channel, which has been related to Na^+^ homeostasis in mice. Indeed, behavioral testing of the Na_v_2.3 null mutant mice (Na_v_2.3^−/−^) indicates that these animals do not show salt aversion under dehydrated conditions and that site-directed transfer of the Na_X_ gene into the SFO restores salt aversion (Watanabe et al., 2000; Hiyama et al., 2004). Further functional studies clearly established Na_X_ as a Na^+^ concentration-sensitive channel (Hiyama et al., 2002). Although progress has been made regarding the role of Na_X_ in Na^+^ homeostasis, including the expression pattern of Na_X_, the precise phenotype of Na_X_-expressing cells in the CVOs remains unclear. The initial study reporting the presence of Na_v_2.3 in the CVOs showed that the gene was highly colocalized with neurofilaments and not with glial fibrillary acidic protein (GFAP) in the SFO and OVLT of the mouse (Watanabe et al., 2000). The functional expression of Na_X_ in neurons was further confirmed using intracellular Na^+^ imaging analysis carried out in dissociated SFO neurons that were immunopositive for the Na_X_ protein (Hiyama et al., 2002). In contrast, Na_X_ was later reported to colocalize with glia-specific glutamate transporter (GLAST) in the SFO and OVLT, thereby indicating that Na_X_ was exclusively expressed in ependymal cells and astrocytes in these organs (Watanabe et al., 2006).

The expression and functional properties of Na_X_ in the brain of the rat show noticeable discrepancies with the mouse. An *in situ* hybridization study has revealed that Na_X_ mRNA is expressed in both the SFO and OVLT in the rat, as well as in the median preoptic nucleus (MnPO; Grob et al., 2004), which is a critical site for the regulation of need-induced Na^+^ ingestion. In that study, Na_X_ mRNA was shown to colocalize with NeuN, a well-recognized neuronal marker, at least in the MnPO. A complementary study performed on dissociated MnPO neurons demonstrated that NeuN immunopositive cells were immunoreactive to an anti-Na_X_ antibody (Tremblay et al., 2011) that had been previously used for Na_X_ detection in several mouse cell types (Knittle et al., 1996; Hiyama et al., 2002). Interestingly, electrophysiological recordings carried out in dissociated MnPO neurons demonstrated that the neuronal Na^+^ sensitivity observed *in situ* (Grob et al., 2004) was attributed to the functional expression of a specific Na^+^ leak channel, which strongly suggests that neuronal Na_X_ is the molecular entity responsible for Na^+^ sensing in the rat MnPO (Tremblay et al., 2011).

The present review of the literature demonstrates that our knowledge of the atypical Na^+^ channel, Na_X_, in brain regions associated with Na^+^ homeostasis is clearly advanced but not yet conclusive. Indeed, these data reveal specific differences in the two rodent species, which are either in terms of brain regions or in terms of cell types that express Na_X_. Regarding the primary role of Na_X_ in sodium sensing in the brain, the presence of this channel in additional brain regions involved in water and electrolyte homeostasis needs to be investigated. Therefore, we developed a specific antibody directed against the interdomain 2–3 region of the α-subunit of the Na_X_ channel. This antibody was used to reveal the distribution of the Na_X_ protein throughout the hypothalamus, the lamina terminalis, and the medulla of rats and mice. Moreover, this study combined specific Na_X_ immunostaining with various fluorescent cell markers to clearly identify the cell types that express Na_X_ in these regions of the rat and mouse brain. The present comparative neuroanatomy study will certainly help to further clarify the distribution of the atypical Na^+^ channel and support its putative role in the control of the hydromineral homeostasis in rats and mice.

## Materials and Methods

### Preparation of anti-Na_X_ antiserum

The polypeptide from the cytoplasmic interdomain 2–3 (ID_2–3_, amino acids 753–917 from *Rattus norvegicus* sequence, accession number NP_113874) was synthesized in the CHUL research centre (Québec, QC, Canada). Briefly, the 495 bp corresponding cDNA sequence (accession number NM_031686) was amplified with the following PCR primers: forward, 5′-GGG*GGATCC*GCTTCATACGATGCTACCACAGAA-3′ and reverse, 5′-GGG*CTCGAG*AAGCTTCGACTTTTCATGTTCATAG-3′, included the restriction enzyme site *Bam*HI and *Xho*I, respectively (underlined sequences).The amplified PCR fragment was cloned in plasmid expression pGEX-4T vector in frame with the glutathione *S*-transferase (GSH-T) tag, and transformed in *E. coli* BL21-Gold (D3) strain. The resulting DNA construction was purified and sequenced on the both strand to check unwanted mutation. Rat Na_X_ fusion protein was induced with 0.1 mM IPTG, 2 h at 37°C and purified on glutathione sepharose 4B column. The purified fusion protein was injected into rabbit for immunization and the antibodies were affinity purified by GenScript Company (GenScript Inc., NJ, USA).

### Animals

Fifteen Wistar male rats (100–150 g) and fifteen C57BL/6 male mice (20–25 g) were purchased from Charles River (Wilmington, MA, USA) 1 week before experimentation. The animals were housed in plastic cages and acclimated to standard laboratory conditions (12 h light/12 h dark cycle; temperature controlled at 23°C). The animals had free access to tap water and regular rat chow (# 2018, Harlan Teklad, Montreal, QC, Canada). The experimental procedures were performed according to the guidelines of the Canadian Council on Animal Care and approved by the animal care committee of our institution (Université Laval).

### Tissue preparation and immunohistochemistry

The rats and mice were deeply anesthetized (ketamine, 87.5 mg/kg and xylazine 12.5 mg/kg solution, i.p. and ketamine, 8.75 mg/kg and xylazine 1.25 mg/kg solution, i.p., respectively) and euthanized by transcardiac perfusion with cold saline (4°C), immediately followed by the perfusion of a fixative solution [4% paraformaldehyde (PFA), pH 7.4; 4°C]. The brains were quickly removed from the skull, post-fixed 6 h in the fixative solution and then immersed for 40 h at 4°C in a sterile phosphate buffer salt (PBS) solution containing 20% sucrose. The brains were frozen on dry ice and mounted on a microtome stage. Coronal sections (30 μm for rats and 20 μm for mice) were collected in a cryoprotectant solution and stored at −20°C. Before use, the brain sections were rinsed in sterile PBS solution.

The primary and secondary antibodies used in this study are listed in Table 1. The free-floating brain sections were incubated 1 h at RT in a blocking solution (PBS solution containing 0.3% Triton X-100, 5% normal goat serum, 1% BSA), and then transferred in PBS solution containing primary antibodies overnight at 4°C. After washing in Tween-containing PBS solution (0.05%), the sections were incubated for 2 h at RT with the secondary antibodies. Finally, the brain sections were mounted onto poly-l-lysine-coated slides, air-dried and coverslipped with Vectashield^®^ mounting medium for fluorescence (Vector Laboratories, CA, USA).

**Table 1:** **List of the primary and secondary antibodies used in the present study**.

Antibody	Animal	Antigen	Dilution	#Cat. or reference	Source
Na_x_	Rabbit, polyclonal	Synthetic peptide of rat Nax amino acids 753–917	1:250		This study
NeuN	Mouse, monoclonal	Purified cells nuclei from mouse brain	1:1,000	MAB377	Millipore, Temecula, CA, USA
GFAP	Mouse, monoclonal	Purified GFAP from pig spinal cord	1:1,000	G3893	Sigma Aldrich, Saint-Louis, MO, USA
Oxytocin-neurophysin	Mouse, monoclonal	Rat oxytocin-associated neurophysin (NP-OT)	1:250	PS-38 (Ben-Barak et al., 1985)	Dr. Harold Gainer (NIH, Bethesda, MD, USA)
Vasopressin-neurophysin	Mouse, monoclonal	Rat vasopressin-associated neurophysin (NP-VP)	1:250	PS-41 (Ben-Barak et al., 1985)	Dr. Harold Gainer (NIH, Bethesda, MD, USA)
Vimentin	Chicken, polyclonal	Recombinant vimentin	1:1,000	AB5733 (Elias et al., 2007)	Millipore, Temecula, CA, USA
Alexa Fluor 488 goat anti-rabbit IgG (H + L)			1:1,000	A-11034	Invitrogen, Burlington, ON, Canada
Alexa Fluor 555 goat anti-mouse IgG (H + L)			1:1,1000	A-21424	Invitrogen, Burlington, ON, Canada
Alexa Fluor 555 goat anti-chicken IgG (H + L)			1:1,000	A-21437	Invitrogen, Burlington, ON, Canada

### Tissue preparation and RT-PCR

Rats and mice were prepared as for tissue preparation for immunofluorescence without tissue fixation step. Briefly Brains were quickly removed from the skull and submerged in ice-cold (2°C) artificial CSF (aCSF) continuously bubbled with a gas mixture (95% O_2_–5% CO_2_) and containing 2 mM KCl, 1 mM CaCl_2_, 3 mM MgCl_2_, 26 mM NaHCO_3_, 1.2 mM NaHPO_4_, 10 mM d-glucose, 200 mM sucrose, pH 7.4. Osmolality was adjusted to 298–300 mOsm/l with mannitol. Coronal brain slices (350 μm thick) were cut with a vibratome (model VT1000S; Leica, Nusloch, Germany) then transferred in aCSF continuously bubbled with a gas mixture (95% O_2_–5% CO_2_). Brain regions of interest were micropunched, transferred in 1.5 ml microtube and freeze in dry ice. Tissue was kept at −80°C until to use.

Total RNA from brain micropunches were extracted and DNase-treated with the PureLink™ RNA Micro kit according to the manufacturer’s instructions (Invitrogen, Burlington, ON, Canada). The concentration was determined by measurement of absorbance at 260 nm with an absorbance reader (model Infinite F200; Tecan, Durham, USA). Reverse transcription (RT) PCR experiments were performed in two steps. First, cDNA synthesis was carried out with the Transcriptor first-strand cDNA synthesis kit (Roche Diagnostic, Quebec, QC, Canada). RT reaction used a mix of random hexamer and oligo-dT primers of the kit with 400 ng of total RNA in a final volume of 20 μl. Three microliters of the first-strand cDNA synthesis reaction was used as template for PCR amplification. PCR fragments were amplified with Maxima^®^ Hot Start *Taq* DNA Polymerase (Fermentas, Burlington, ON, Canada) and primers: MOUD66: 5′-ACTGTGTTCCGAATCCTCTG-3′/MOUD59: 5′-TCATGTCTCCATACTCCAGG-3′ for Na_X_, and MOUD82: 5′-GTCTTCACTACCATGGAGAAGG-3′/MOUD83: 5′-TCATGGATGACCTTGGCCAG-3′ for GAPDH (Jia et al., 2010). The PCR program to amplify the 890 bp for Na_X_ and 200 bp for GAPDH products was as follows: one hold at 94°C for 5 min followed by 35 cycles of 94°C for 30 s, 58°C for 30 s, and 72°C for 60 s, and were visualized on a 2% (w/v) agarose gel.

### Image analysis

High-resolution images were collected on a i90 Nikon fluorescence microscope (Nikon Canada Inc., Mississauga, ON, Canada) coupled to a Hamamatsu 1394 ORCA-285 monochrome camera and exploited by Simple PCI software version 5.3.0.1102 (Compix Inc. Imaging Systems, PA, USA). Finally, all images were adjusted for brightness and contrast using Adobe Photoshop, and the figures were assembled in Adobe Illustrator.

## Results

### Design, synthesis, and purification of the immunogenic Na_X_ peptide

**Figure 1:**
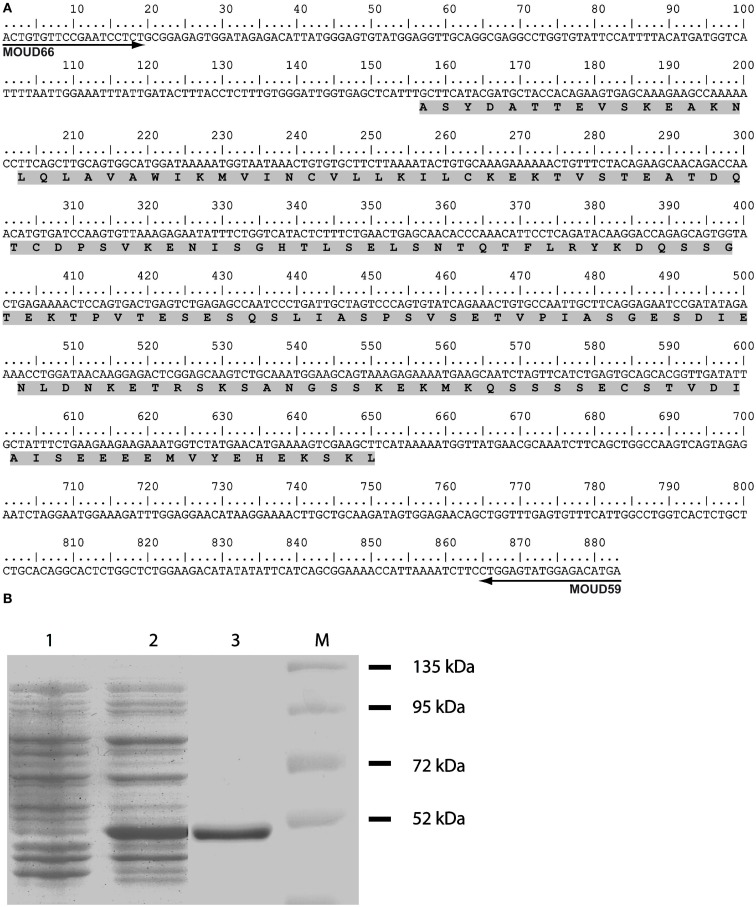
**Synthetic peptide used to produce the anti-Na_X_ antibody**. **(A)** Representation of the partial nucleotide sequence of the Na_X_-ID_2–3_. The nucleotide sequence corresponds to the nucleic acid targeted for RT-PCR experiment carried out with the primers MOU66 and MOU59. The sequence of the synthetic peptide used for the rabbit immunization is highlighted in gray. **(B)** 12% acrylamide SDS-PAGE analysis characterizes the purification of the Na_X_-GST fusion protein. Lanes contain non-induced crude lysate (1), 0.1 mM IPTG-induced crude lysate (2) and purified 49 kDa Na_X_-GST fusion protein (3). M, protein size marker.

The aim of the present study was to determine the distribution and phenotype of the cells that express the Na_X_ channel in the rat and mouse brain. Accordingly, we developed a specific antibody directed against the cytoplasmic loop linking domains 2 and 3 (ID_2–3_) of the Na_X_ channel. This region was targeted because protein sequence analysis revealed that ID_2–3_ is unique among the voltage-gated Na^+^ channel family members. Indeed, the targeted 165 amino acids sequence (Figure 1A) shares a low percentage of identity (ranging 13–42%) with the other voltage-gated sodium channels (Na_v_1.1–Na_v_1.9, Table 2). Interestingly, this protein sequence identified in the rat shares a high percentage of identity (87%) with the protein sequence identified in the mouse. Taken together, we assumed that the polyclonal antibody produced from the ID_2–3_ immunogenic peptide was Na_X_ specific and could be used to detect Na_X_ expression in both the mouse and rat.

**Table 2:** **Percentage of identity between the rat Na_X_ antibody epitope used in this study, the mouse Na_X_ and the voltage-gated Na_V_ sodium channels**.

Name	Species	Acc. Num.		% identity
Na_X_	Mouse	NP_033161		87
Na_V_ 1.1	Rat	P04774	35	
	Mouse	NP_061203		38
Na_V_ 1.2	Rat	P04775	37	
	Mouse	NP_001092768		37
Na_V_ 1.3	Rat	P08104	34	
	Mouse	NP_061202		35
Na_V_ 1.4	Rat	AAA41682	25	
	Mouse	NP_573462		30
Na_V_ 1.5	Rat	P15389	14	
	Mouse	NP_067519		7
Na_V_ 1.6	Rat	AAC42059	29	
	Mouse	NP_035453		29
Na_V_ 1.7	Rat	AAB80701	42	
	Mouse	Q62205		42
Na_V_ 1.8	Rat	Q63554	19	
	Mouse	Q6QIY3		10
Na_V_ 1.9	Rat	NP_062138	13	
	Mouse	NP_036017		8

The ID_2–3_ immunogenic peptide was produced in *E. coli* and expressed using the recombinant plasmid pGEX-Na_X_-ID_2–3_. Na_X_-GST fusion protein expression was induced using IPTG and purified by glutathione affinity chromatography as described in Section “Materials and Methods.” The quality of the protein purification was evaluated using SDS-PAGE (Figure 1B).

### Antibody production and specificity

**Figure 2:**
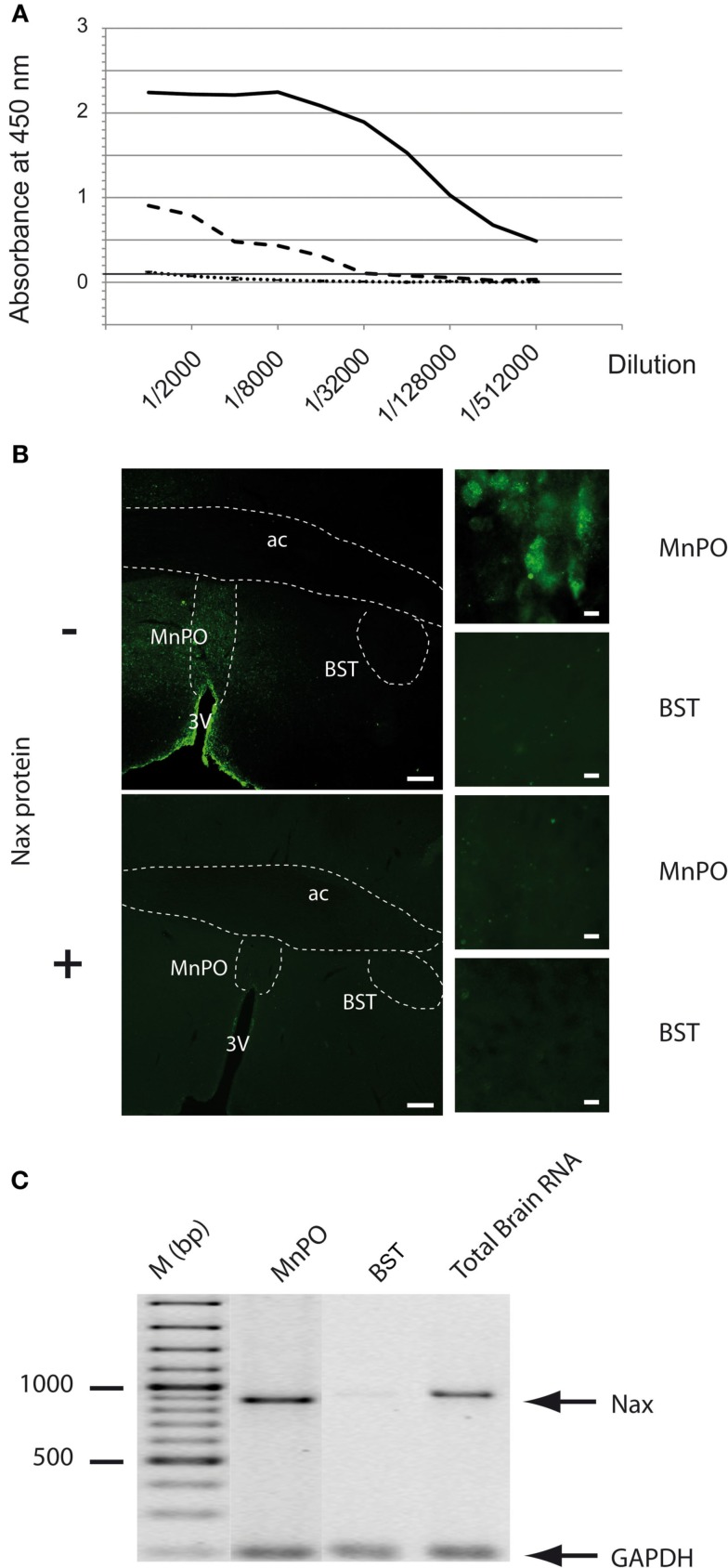
**Specificity and validation of the anti-Na_X_ antibody**. **(A)** The immune serum collected from the rabbits was assessed by ELISA and revealed the presence of the Na_X_ protein and not the GST tag. The microplate was coated with the Na_X_ peptide (1 μg/well), incubated with pre-immune serum (dotted line), or double purified immune serum (black line: antibody titer >>1/512,000). To control purification efficiency, ELISA was also performed on GST protein with double purified immune serum (dashed line). The rabbit antibodies were revealed with goat anti-rabbit IgG, HRP conjugated secondary antibody and the absorbance was read at 450 nm. **(B)** The purified anti-Na_X_ antibody (antibody concentration 1/250) was tested for immunohistochemical staining without (−) or with (+) preabsorption with the antigen peptide (50 μg/ml). Fluorescent immunostaining in the MnPO nucleus served as a positive control, whereas absence of immunostaining in the BST nucleus is a negative control. Scale bar: 200 μm (low magnification), 10 μm (high magnification). **(C)** The immunohistochemical staining was correlated with the presence of Na_X_ mRNA revealed by RT-PCR experiment carried out on micropunched region of the rat MnPO and BST. Note that the expected size of amplified products was 883 bp for Na_X_ and 200 bp for GAPDH (housekeeping gene). Commercial rat brain total RNA (Clontech Laboratories Inc.) was used as a positive control.

The purified Na_X_-GST protein was used to immunize 2 rabbits to produce the polyclonal anti-Na_X_ antibody. The purified Na_X_-GST protein was injected four times in 2 months. The rabbits were bled 1 week after the fourth injection of the immunogenic peptide and the immune serum was purified in two steps. The first purification step was to eliminate the antibodies that did not bind the Na_X_-GST protein, which was then followed by a step to eliminate the antibodies that solely bound the GST-tag of the immunogenic peptide. Validation of the anti-Na_X_ antibody was achieved with several tests. First, the ability of the purified antibodies to bind the Na_X_ protein was performed using an ELISA test (results provided by GenScript Inc., Figure 2A). This test demonstrated that only the purified antibodies bound the Na_X_ protein, whereas the pre-immune serum did not bind the immunogenic peptide (negative control). Note that the antibody titer was defined as the maximal dilution giving an OD_450 nm_ signal greater than 0.1 and was calculated at a 1/512,000 dilution for the antibody batch used in the present study. Subsequently, the specificity of the anti-Na_X_ antibody was verified *in situ* with immunohistochemical staining. Using thin (30 μm) brain slices, we evaluated Na_X_ immunostaining in the rat MnPO (positive control) and BST (negative control; Figure 2B, upper panels). As expected, fluorescent immunostaining was restricted to the MnPO, which is known to express both Na_X_ mRNA and protein (Grob et al., 2004; Tremblay et al., 2011). In contrast, no fluorescent staining was observed in the BST, which has been shown to be devoid of Na_X_ mRNA as previously reported (Grob et al., 2004). Preadsorption carried out with increasing concentrations of the immunizing peptide (0–200 μg/ml) indicated that the fluorescent immunostaining in the MnPO could be totally abolished when the anti-Na_X_ antibody was pre-incubated with the immunizing peptide (50 μg/ml; Figure 2B, lower panels). Preadsorption carried out with an excess of GST protein (200 μg/ml) did not interfere with immunostaining in the MnPO (data not shown). Finally, the immunostaining was correlated with the presence or the absence of RT-PCR products isolated from the MnPO and BST, respectively (Figure 2C).

Together, this series of experiments led to the production and validation of a polyclonal rabbit anti-Na_X_ antibody. This antibody is highly specific toward the rat and mouse Na_X_ ID_2–3_.

### Mapping of the Na_X_ protein shows noticeable differences in the rat and mouse brain

To examine the specificity in the Na_X_ distribution in the rat and mouse brain, the entire brains of these rodents were sliced in thin sections and the regions of interest, which included all of the CVOs, the MnPO and the supraoptic and paraventricular nucleus (SON, PVN), were incubated with the anti-Na_X_ antibody. In addition, fluorescent Na_X_ immunostaining was combined with various cell markers to identify the cellular phenotype that expressed the Na_X_ channel. Thus, Na_X_ immunostaining was performed in combination with anti-NeuN, anti-GFAP, or anti-vimentin immunohistochemistry to identify neurons, glial cells or ependymal cells and tanycytes, respectively. Immunostaining was also performed with oxytocin and vasopressin-associated neurophysin to distinguish between oxytocin and vasopressin-expressing magnocellular neurons.

Although rats and mice are phylogenetically close animals, our anatomical analysis revealed noticeable differences in terms of patterns and cell types in the brain that expressed Na_X_.

### Na_X_ immunofluorescence in the circumventricular organs and in the median preoptic nucleus

**Figure 3:**
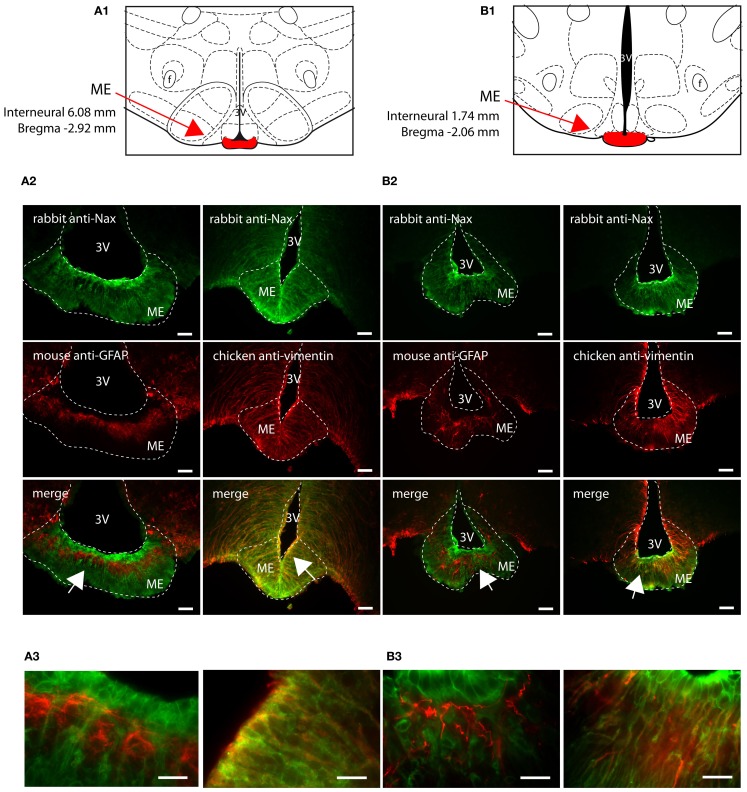
**Distribution of Na_X_ immunostaining in the median eminence of the rat and mouse**. Schematic illustration of the median eminence (ME) of the rat **(A1)** and of the mouse **(B1)**. ME was filled in red for better identification. f, fornix; 3V, third ventricle. Representative picture of the fluorescent Na_X_ immunostaining (green) was obtained from the rat **(A2)** and from the mouse **(B2)**. The cell type expressing Na_X_ was identified using anti-GFAP and anti-vimentin fluorescent immunostaining (red). Scale bar: 100 μm. The arrow head points the top-right, the bottom-right, and the bottom-left corner of the inset, which represents a high magnification zone of the ME in the rat **(A3)** and in the mouse **(B3)**; scale bar: 20 μm. Note that Na_X_ staining is present in vimentin positive cells.

**Figure 4:**
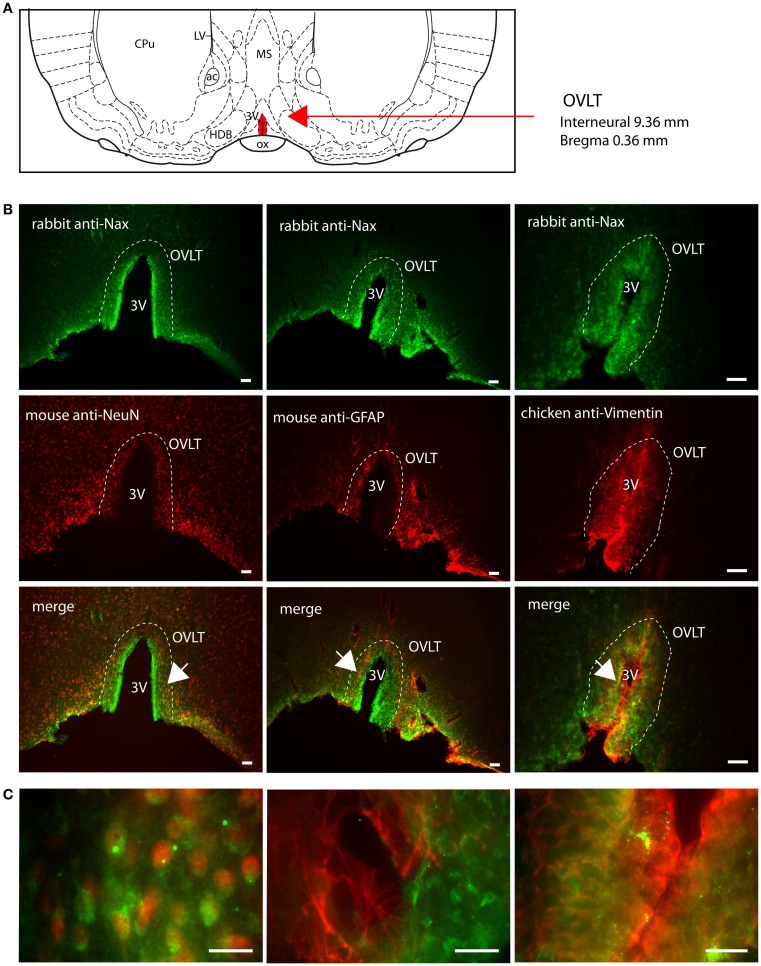
**Distribution of Na_X_ immunostaining in the vascular organ of the lamina terminalis of the rat**. **(A)** Schematic illustration of the vascular organ of the lamina terminalis (OVLT) in the ventral forebrain. This small structure is filled in red for better visualization. ac, anterior commissure; CPu, caudate putamen; HDB, horizontal limb of the diagonal band; LV, lateral ventricle; MS, medial septum; ox, optic chiasm; 3V, third ventricle. **(B)** Representative picture of the fluorescent Na_X_ immunostaining (green) was obtained from the rat. The OVLT region is delimited by the dashed line. 3V, third ventricle. The cell type expressing Na_X_ was identified using anti-NeuN, anti-GFAP, and anti-vimentin fluorescent immunostaining (red); scale bar: 100 μm. **(C)** High magnification pictures of the staining merge; scale bar: 20 μm. The arrow head points the top-right corner (left panel) or the top-left corner (middle and right panel) of the enlarged area. Note that Na_X_ staining is present in NeuN expressing cells and in vimentin positive cells.

**Figure 5:**
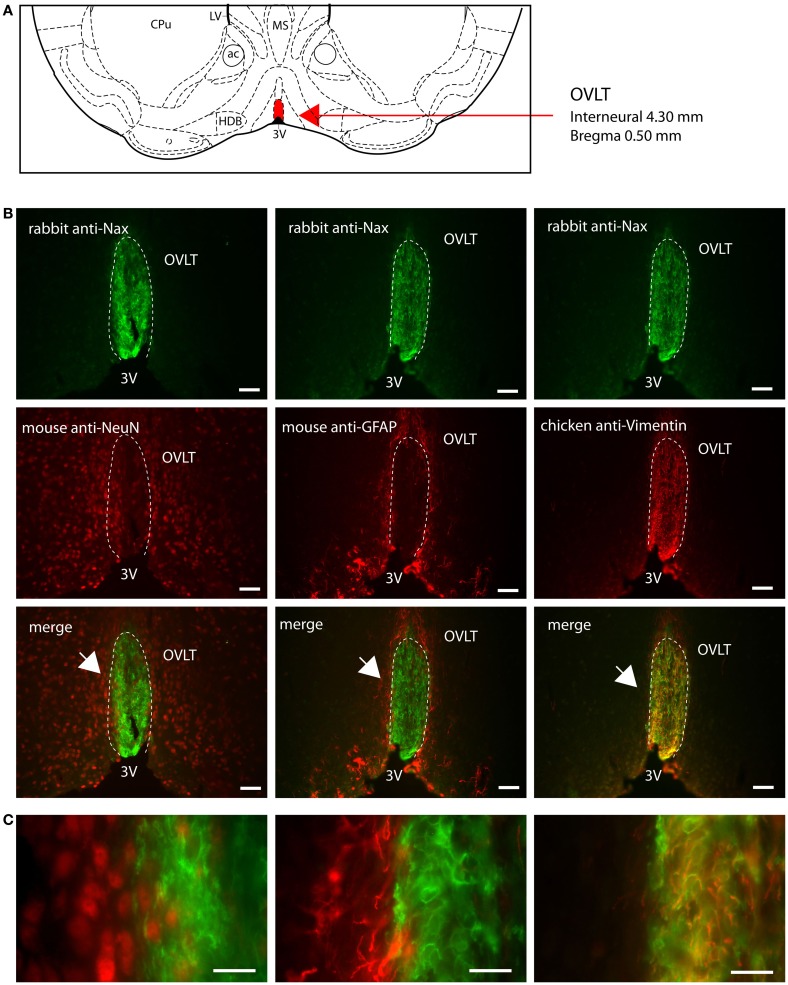
**Distribution of Na_X_ immunostaining in the vascular organ of the lamina terminalis of the mouse**. **(A)** Schematic illustration of the vascular organ of the lamina terminalis (OVLT) in the ventral forebrain of the mouse. The OVLT appears in red for better visualization. ac, anterior commissure; CPu, caudate putamen; HDB, horizontal limb of the diagonal band; LV, lateral ventricle; MS, medial septum; 3V, third ventricle. **(B)** Representative picture of the fluorescent Na_X_ immunostaining (green) was obtained from the mouse. The OVLT region is delimited by the dashed line. 3V, third ventricle. The cell type expressing Na_X_ was identified using anti-NeuN, anti-GFAP, and anti-vimentin fluorescent immunostaining (red); scale bar: 50 μm. **(C)** High magnification pictures of the staining merge; scale bar: 20 μm. The arrow head points the top-left corner of the enlarged zone. Na_X_ staining is only present in vimentin positive cells.

**Figure 6:**
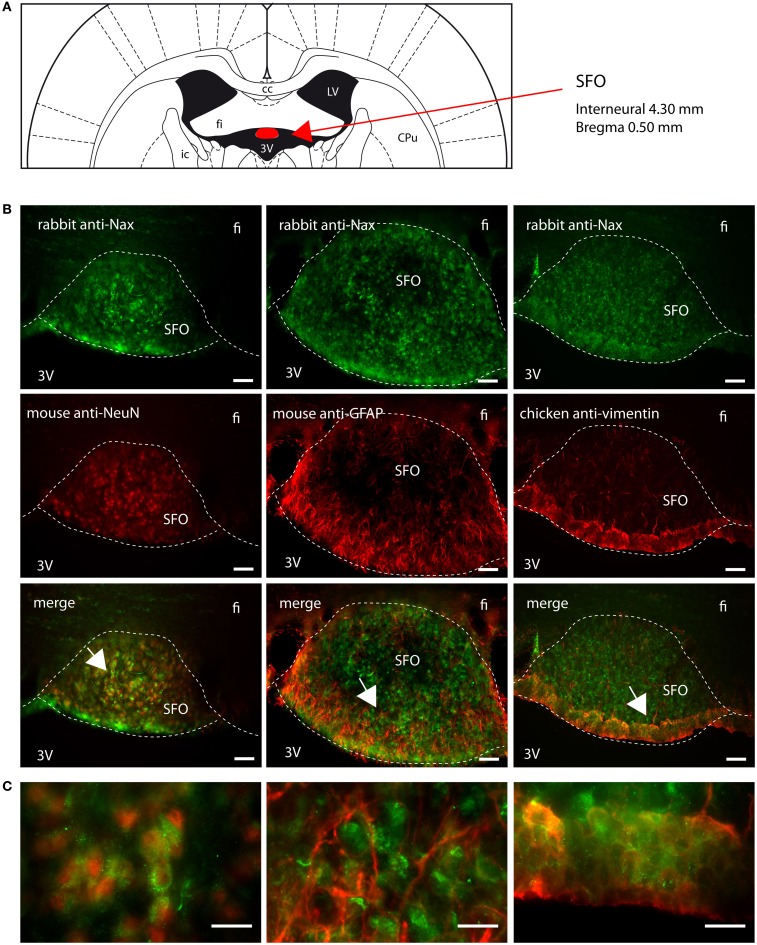
**Distribution of Na_X_ immunostaining in the subfornical organ of the rat**. **(A)** Schematic illustration of the subfornical organ (SFO) in the rat brain. The SFO appears in red for better visualization. cc, corpus callosum; CPu, caudate putamen; fi, fimbria hippocampus; ic, internal capsule; LV, lateral ventricle; 3V, third ventricle. **(B)** Representative picture of the fluorescent Na_X_ immunostaining (green) was obtained from the rat. The cell type expressing Na_X_ was identified using anti-NeuN, anti-GFAP, and anti-vimentin fluorescent immunostaining (red); scale bars: 50 μm. **(C)** Inset representing high magnification area of the SFO; scale bar: 20 μm. The enlarged zone is pointed by the arrow head (top-left corner of the inset). Na_X_ staining is present in NeuN positive cells in the core of the SFO and in vimentin positive cells in the cell layer boarding the third ventricle.

**Figure 7:**
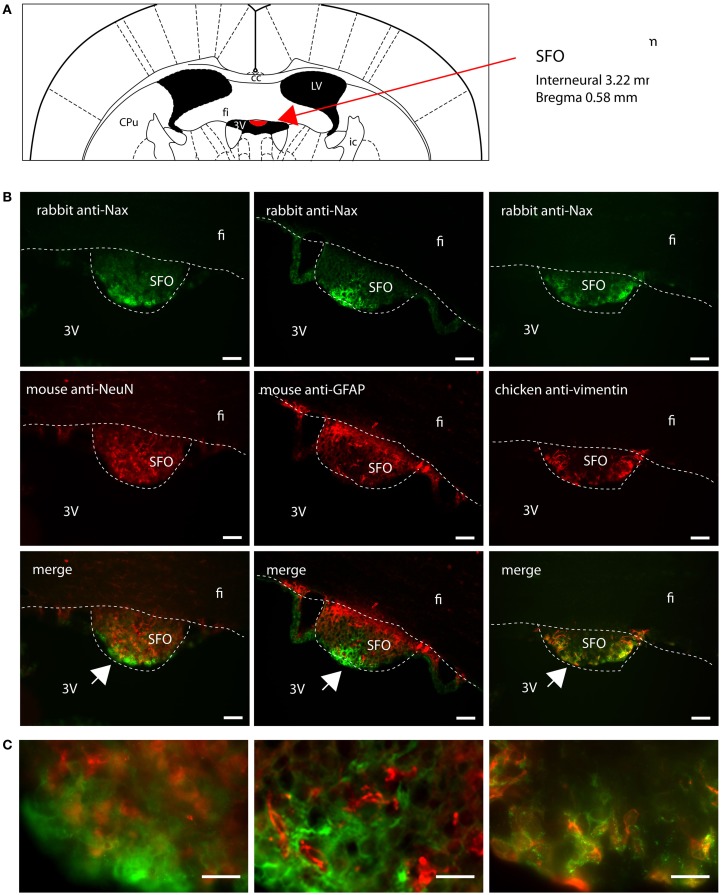
**Distribution of Na_X_ immunostaining in the subfornical organ of the mouse**. **(A)** Schematic illustration of the subfornical organ (SFO) in the mouse brain (red filling). CPu, caudate putamen; fi, fimbria hippocampus; ic, internal capsule; LV, lateral ventricle; 3V, third ventricle. **(B)** Representative picture of the fluorescent Na_X_ immunostaining (green) was obtained from the mouse. The cell type expressing Na_X_ was identified using anti-NeuN, anti-GFAP, and anti-vimentin fluorescent immunostaining (red); scale bars: 50 μm. **(C)** Inset representing high magnification zone of the SFO; scale bar: 20 μm. The detailed zone is pointed by the arrow head (bottom-left corner of the inset). Na_X_ staining is only present in the vimentin positive cells boarding the third ventricle.

Na_X_ immunostaining in both the rat and the mouse was detected in the CVOs, the OVLT, the SFO, the ME, and the area postrema (AP). The pattern and cellular phenotype for Na_X_ expression in both rats and mice showed similarities only in two CVOs, which were the ME and the AP. In the ME, Na_X_ immunostaining was essentially observed in the ventral portion of the wall of the third ventricle and in the radial processes penetrating the neuropil. Na_X_ immunostaining colocalized with vimentin immunostaining only (Figure 3; Table 3). Intriguingly, Na_X_ immunostaining was associated with none of the cell markers in the AP (Table 3). However, the clear regionalization of Na_X_ immunostaining (central, lateral, and ventral zone), of vimentin staining (funiculus separans, fs) and of GFAP staining (inverted pyramidal region extending from the fs to the central canal) ruled out possible colocalization of Na_X_ immunostaining with tanycytes or glial cells in the rat and mouse AP. In the other CVOs, Na_X_ cellular localization was strongly divergent (Table 3). Indeed, in the rat OVLT, Na_X_ immunostaining was dense in the cell layers boarding the third ventricle and in individual cells of the lateral periventricular tissue. Na_X_ immunostaining colocalized with NeuN immunostaining in the lateral part of the OVLT and with vimentin immunostaining lining the third ventricle (Figure 4). In the mouse OVLT, Na_X_ immunostaining was confined to a dense fiber network and only colocalized with vimentin immunostaining (Figure 5). Na_X_ immunostaining was not colocalized with NeuN or GFAP immunostaining. The distribution pattern of the Na_X_-expressing cells was also different in the SFO of the two rodents. In the rat SFO, Na_X_ immunostaining was present throughout the organ with a clear staining of the ependymal cell layers boarding the ventricular surface. Na_X_ immunostaining colocalized with NeuN positive cells in both the core and periphery of the SFO, as well as with vimentin immunostaining in the ependymal cell layers (Figure 6). In the mouse SFO, Na_X_ immunostaining was present in clusters boarding the ventricular surface. Na_X_ immunostaining colocalized with vimentin immunostaining only (Figure 7). Overall, these results indicate that Na_X_ is expressed in neurons and ependymocytes in the rat OVLT and SFO, and only in ependymocytes in the mouse organs (Table 3).

**Table 3:** **Colocalization of Na_x_ immunostaining with fluorescent cell markers in the CVOs and the MnPO**.

	Rat				Mouse			
	Na_x_	Na_x_ + NeuN	Na_x_ + GFAP	Na_x_ + Vimentin	Na_x_	Na_x_ + NeuN	Na_x_ + GFAP	Na_x_ + Vimentin
OVLT	+	+	−	+	+	−	−	+
SFO	+	+	−	+	+	−	−	+
EM	+	−	−	+	+	−	−	+
AP	+	−	−	−	+	−	−	−
MnPO	+	+	−	−	−	−	−	−

*AP, area postrema; ME, median eminence; MnPO, median preoptic nucleus; OVLT, vascular organ of the lamina terminalis; SFO, subfornical organ*.

**Figure 8:**
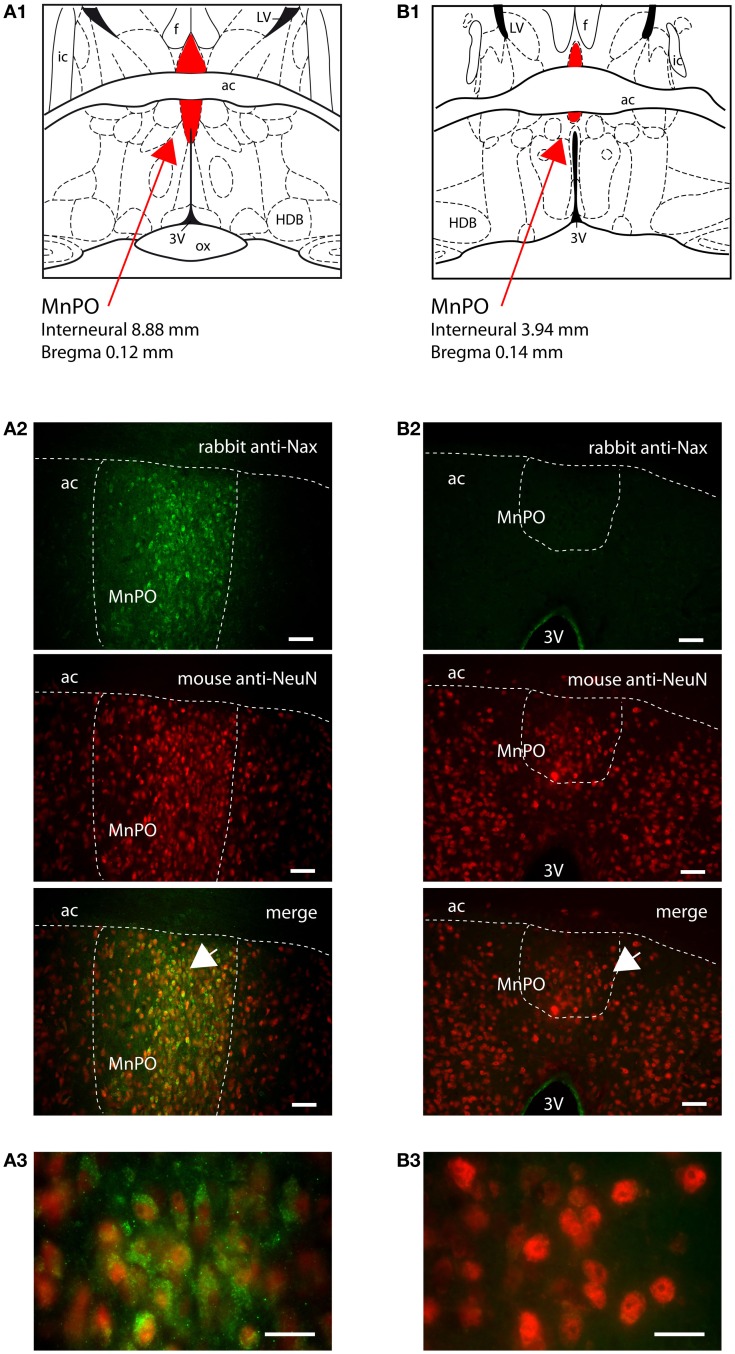
**Na_X_ immunostaining was restricted to the median preoptic nucleus of the rat**. **(A1)** Schematic illustration of the median preoptic nucleus (MnPO) of the rat. The nucleus appears in red for better visualization. Note that the ventral part is separated from the dorsal part of the nucleus by the anterior commissure (ac). f, fornix; HDB, horizontal limb of the diagonal band; ic, internal capsule; LV, lateral ventricle; ox, optic chiasm; 3V, third ventricle. **(A2)** Representative picture of fluorescent Na_X_ immunostaining (green) was obtained from the ventral part of the rat MnPO; scale bar: 50 μm. The cell type expressing Na_X_ was identified using anti-NeuN fluorescent immunostaining (red). The arrow head points the top-right corner of the inset that represents a high magnification zone of the ventral MnPO; scale bar: 20 μm. Note the colocalization of the fluorescent Na_X_ and NeuN immunostaining. **(B1)** Schematic illustration of the mouse MnPO (red filling). **(B2)** Representative picture showing that fluorescent Na_X_ immunostaining was restricted to the cell layer boarding the third ventricle in the mouse; scale bar: 50 μm. The arrow head points the top-right corner of the inset that represents a high magnification zone of the ventral MnPO in the rat **(A3)** and in the mouse **(B3)**; scale bar: 20 μm.

The MnPO lies in a strategic position in order to monitor CSF ion composition and, thus, CSF osmolality. This functional role results from the absence of tanycytes and their tight junctions at the CSF–MnPO interface and from the presence of ependymal ciliated cells that permit passive diffusion of ions from the CSF. In the MnPO, the Na_X_ immunostaining was observed in individual cells homogeneously distributed in the nucleus. Na_X_ immunostaining solely colocalized with NeuN immunostaining in the rat (Table 3; Figure 8A). Interestingly, the mouse MnPO was characterized by the absence of Na_X_ immunostaining (Figure 8B).

Together, the present data demonstrate noticeable differences between the rat and mouse in the distribution pattern and the cell types expressing Na_X_ protein in the brain regions involved in the detection of osmolarity and Na^+^ concentration.

### Na_X_ immunofluorescence in the supraoptic and paraventricular nuclei

**Figure 9:**
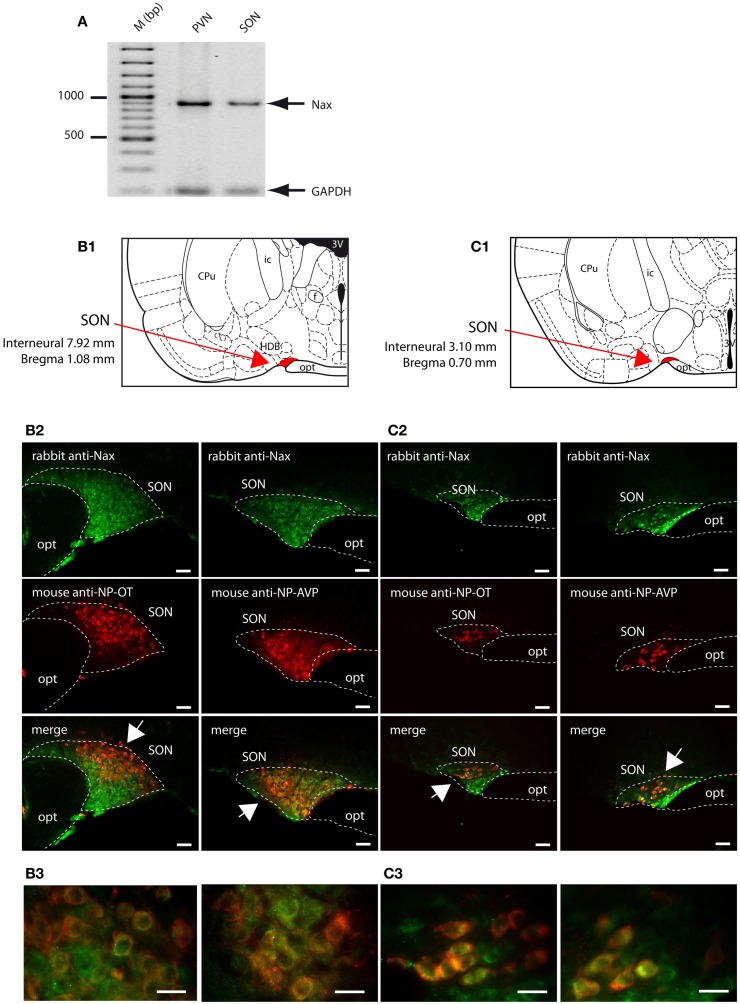
**Na_X_ is expressed in the magnocellular neuroendocrine cells of the rat and mouse supraoptic nucleus**. **(A)** Na_X_ mRNA expression was revealed by RT-PCR experiment carried out on micropunched region of the SON and PVN. Note that the expected size of the amplified products was 883 bp for Na_X_ and 200 bp for GAPDH (housekeeping gene). Schematic illustration of the SON in the rat **(B1)** and mouse **(C1)**. The SON appears in red for better visualization. Representative distribution of fluorescent Na_X_ immunostaining (green) in the magnocellular neurons of the SON obtained from the rat **(B2)** and mouse **(C2)**. The neurochemical content of the cells expressing Na_X_ was identified with the anti-oxytocin neurophysin and the anti-vasopressin neurophysin fluorescent immunostaining (red); scale bars: 50 μm. The arrow head points the top-right or the bottom-left corner of the inset, which represents a high magnification zone of the rat SON **(B3)** and the mouse SON **(C3)**; scale bar: 20 μm. Not that Na_X_ immunostaining was present in both vasopressinergic and oxytocinergic magnocellular cells in the rat and mouse.

**Figure 10:**
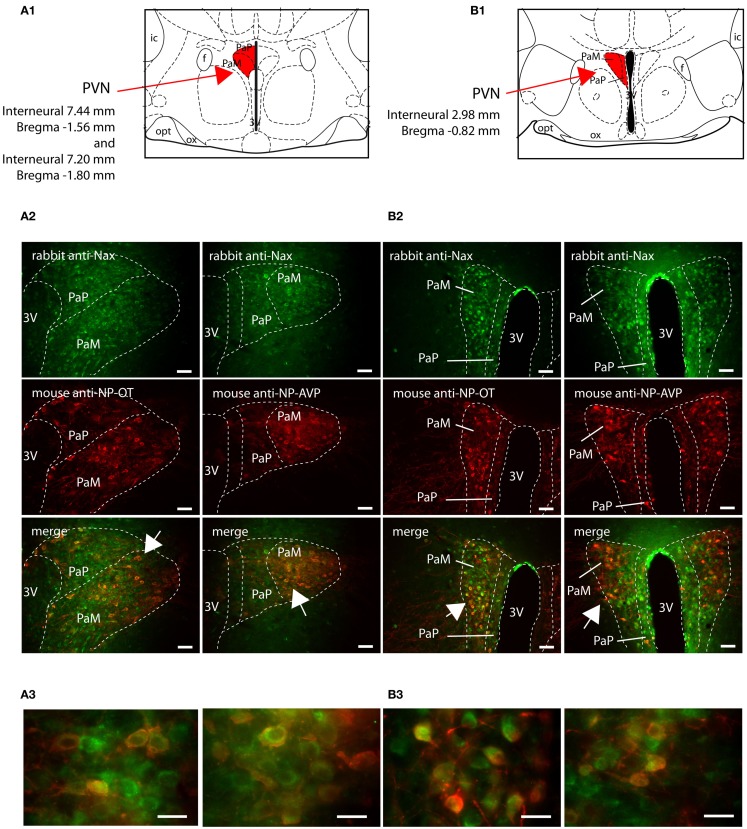
**Na_X_ is expressed in the magnocellular and parvocellular neuroendocrine cells of the rat and mouse paraventricular nucleus**. Schematic illustration of the representative PVN sections in the rat **(A1)** and in the mouse **(B1)**. The PVN divisions appear in red for better visualization. Representative distribution of fluorescent Na_X_ immunostaining (green) in the magnocellular part (PaM) and in the parvocellular part (PaP) of the PVN obtained from the rat **(A2)** and mouse **(B2)**. The neurochemical content of the cells expressing Na_X_ was identified with the anti-oxytocin neurophysin and the anti-vasopressin neurophysin fluorescent immunostaining (red); scale bars: 50 μm. The arrow head points the top-right, the bottom-right, and the bottom-left corner of the inset, which represents a high magnification zone of the PVN in the rat **(A3)** and in the mouse **(B3)**; scale bar: 20 μm. Not that NaX immunostaining was present in both vasopressinergic and oxytocinergic magnocellular cells in the rat and mouse.

The SON and the PVN of the hypothalamus are the primary regions responsible for the control of hydromineral balance, which is because the magnocellular neurons in both the SON and PVN synthesize antidiuretic hormone (vasopressin) and antinatriuretic hormone (oxytocin) in rodents. Na_X_ immunostaining in the SON and the PVN was comparable between the two species, and it was primarily observed in the magnocellular neurons (Table 4). Na_X_ immunostaining in these nuclei was clearly supported by a positive Na_X_ RT-PCR signal in the micropunched tissues from the SON and PVN (Figure 9A). To further discriminate which population of magnocellular neurons expressed the Na_X_ channel, Na_X_ immunofluorescence was combined with anti-neurophysin associated vasopressin (VP–NP) and oxytocin (OT–NP) immunofluorescence (the anti-VP–NP and anti-OT–NP antibodies were provided by Dr. H. Gainer (NIH, Bethesda, MD, USA). These results show that Na_X_ immunostaining colocalizes with the magnocellular vasopressinergic and oxytocinergic neurons in the SON (Figures 9B,C) and in the PVN (Figures 10A,B). Moreover, most of the neuronal population showing Na_X_ immunostaining in the parvocellular part of the PVN was not immunofluorescent for VP–NP or OT–NP (Figure 10).

**Table 4:** **Colocalization of Na_X_ immunostaining with fluorescent cell markers in the SON and the PVN**.

	Rat				Mouse			
	Na_x_	Na_x_ + NeuN	Na_x_ + GFAP	Na_x_ + Vimentin	Na_x_	Na_x_ + NeuN	Na_x_ + GFAP	Na_x_ + Vimentin
SON magnocellular	+	+	−	−	+	+	−	−
PVN magnocellular	+	+	−	−	+	+	−	−
PVN parvocellular	+	+	−	−	+	+	−	−

## Discussion

The present study used a Na_X_ antibody specifically designed against the ID_2–3_ region of the channel’s α-subunit in order to compare the mouse and rat’s expression patterns among the brain areas involved in the control of hydromineral balance. In addition, double immunohistochemical staining was performed to characterize the phenotype of the cells that expressed Na_X_ in both rodent brains. Our results indicate that the expression pattern of Na_X_ and the cell type distribution show similarities between the rat and mouse, as well as noticeable differences. In these two rodents, Na_X_ was clearly present in the primary regions involved in hydromineral homeostasis, which are the SFO, OVLT, and the ME. Interestingly, Na_X_ was also found in the AP, the SON, and the PVN of these two species, thereby supporting the claim that this channel is involved in the osmoregulatory process and control of salt appetite. Na_X_ was strongly expressed in the rat but not in the mouse MnPO. However, the most noticeable differences observed between the rat and mouse concerned the phenotype of the cells expressing Na_X_ in the forebrain CVOs. Indeed, in the rat, Na_X_ is present specifically in neurons of the SFO, OVLT, and MnPO, as well as in the ependymocytes lining the two CVOs. In the mouse, Na_X_ is only expressed in the ependymocyte layers boarding the SFO and the OVLT. This comparative anatomical study was carried out with a highly sensitive anti-Na_X_ antibody, which identified the pattern of Na_X_ expression and clarified the specificity of the Na_X_-expressing cell types in the brain regions that are involved in sodium detection in the rat and mouse.

### Validation of the polyclonal anti-Na_X_ antibody targeting the ID_2–3_ region of the Na^+^ channel

To compare the expression pattern and the phenotype of cells expressing Na_X_ in the rat and mouse brain, we produced an antibody against the ID_2–3_ region of the rat Na_X_ protein. This region is highly conserved between the rat and mouse but shows weak homology with other voltage-gated sodium channels. A mandatory step in validating this anti-Na_X_ antibody was to assess the purification level and the specificity toward the Na_X_ channel. We showed that the polyclonal anti-Na_X_ antibody specifically bound the immunogenic peptide without interference from the GST protein. The anti-Na_X_ antibody labeled the rat MnPO nucleus, which has been previously described as being a Na_X_-positive brain nucleus (Grob et al., 2004; Tremblay et al., 2011). Furthermore, this labeling was reduced to background levels after pre-incubation with an excess of the immunogenic peptide. Importantly, Na_X_ immunostaining was correlated with Na_X_ mRNA expression in Na_X_-positive and Na_X_-negative regions of the rat brain (MnPO and BST, respectively; Grob et al., 2004).

Overall, multiple tests validated the specificity of the antibody against the ID_2–3_ region of the Na_X_ channel. Thus, this tool appears appropriate for evaluating Na_X_ distribution and for identifying the cell types expressing this distinct Na^+^ channel.

### Na_X_ immunostaining in the CVOs and the MnPO

The function of the Na_X_ channel has been well characterized in the context of Na^+^ homeostasis in the forebrain CVOs of the mouse (Hiyama et al., 2002; Watanabe et al., 2003; Shimizu et al., 2007) and in the rat MnPO (Grob et al., 2004; Tremblay et al., 2011), where Na_X_ constitutes the molecular basis of the brain’s ability to sense Na^+^. Here, we show that Na_X_ distribution was highly similar in the two rodents because Na_X_ immunostaining was clearly observed in all the CVOs of the rat and mouse. However, a noticeable difference was the presence of Na_X_ immunostaining in the rat MnPO, whereas this nucleus was Na_X_-immunonegative in the mouse. This Na_X_ distribution pattern was validated under similar experimental conditions for the two rodents, which demonstrates the presence of Na_X_ throughout the lamina terminalis of the rat and not just in the MnPO. Moreover, the specific Na_X_ expression pattern in the MnPO of the rat may contribute to a more complex organization of the sodium sensing network in this animal and may represent an additional detection site for CSF sodium levels. Note that the absence of Na_X_ immunostaining in the mouse MnPO has a direct physiological relevance: patch-clamp recordings of dissociated mouse MnPO neurons revealed the absence of membrane depolarization during the occurrence of hypernatriuric artificial CSF (Tremblay et al., 2011).

In addition to the differential Na_X_ distribution between these two rodents, the primary difference between the rat and mouse was in regard to the cell types displaying Na_X_ immunostaining in the CVOs. In the rat, Na_X_ was colocalized with NeuN, a neuronal marker, in the lateral periventricular part of the OVLT and in the core of the SFO. Na_X_ was also colocalized with vimentin, a marker of ependymocytes and tanycytes (Dahl, 1981; Schnitzer et al., 1981; Pixley, 1984; Petito et al., 1990), in the cell layers lining the third ventricle in the OVLT, SFO, and ME. In the mouse, Na_X_ immunostaining was mainly, if not exclusively, colocalized with vimentin in the OVLT, SFO, and ME. This result adds further support to previous data reporting the presence of Na_X_ in glial processes in the mouse CVOs (Watanabe et al., 2006). In that study, Na_X_ immunostaining was reported to colocalize with GLAST immunostaining, which is a glutamate transporter expressed in glial cells. However, GLAST is expressed in both astrocytes and tanycytes (Shibata et al., 1997; Berger and Hediger, 2001), and might not allow to distinguish between the two cell types. In the present study, there was clear colocalization of vimentin and Na_X_ immunostaining in the SFO, OVLT, and ME. Na_X_ immunostaining was also colocalized with GLAST positive cells in the mouse SFO (data not shown), like previously observed (Watanabe et al., 2006). Although vimentin and GFAP have been reported in tanycytes, the GFAP content is very low and tanycytes generally appear vimentin immunopositive and GFAP negative in the mediobasal hypothalamus and CVOs, like the ME and the subcommissural organ (Chouaf et al., 1989; Chauvet et al., 1995, 1998). Na_X_ immunostaining in the SFO, OVLT, and ME, concomitant with the absence of colocalization of GFAP, indicates that the Na_X_ channel is expressed in the ependymocytes and/or tanycytes lining the third ventricle in the rat and mouse. This observation was puzzling at a first glance because Na_X_ gene was isolated from cultured rat astroglia (Gautron et al., 1992). The cell cultures used in that study were prepared from 1-day-old pups and in the rat, tanycytes are generated during the late pregnancy and the first postnatal day (Rodriguez et al., 2005). Because, the radial glial cells are transformed into astrocytes and also differentiated into tanycytes during the perinatal period, Na_X_ RNA might have been isolated from a mixed population of glial cells. Alternatively, the presence of a subpopulation of astrocytes that express vimentin, GLAST and GFAP in the SFO and OVLT might be possible. This putative population is however weak in the forebrain CVOs because most of the GFAP immunostaining identified stellate cells and processes that were not Na_X_ immunopositive.

The expression of the Na_X_ channel in the neurons, ependymocytes and tanycytes raises the question of the degree of complexity for sodium sensing in the rat. In addition to the unique Na_X_ expression in the rat MnPO neurons, it is conceivable that the neuronal populations of the SFO and OVLT encode sodium levels in a similar way (Grob et al., 2004; Tremblay et al., 2011). In fact, the neuronal mechanism of sodium sensing in the MnPO allows the transduction of both hypo- and hypernatriuric signals into membrane hyperpolarization and depolarization, respectively (Grob et al., 2004; Tremblay et al., 2011). Therefore, neuronal Na_X_ in the rat CVOs may transduce sodium levels ranging below and above the sodium set point into distinct firing patterns. In addition, a second mechanism involving ependymocytes and/or tanycytes may lead to lactate release and a resulting change in the excitability of neighboring neurons, which has been reported in the mouse SFO (Shimizu et al., 2007). This mechanism will be engaged under hypernatriuric conditions only and may possibly bolster the initial neuronal signaling.

Interestingly, our results show that Na_X_ is expressed in the AP of both the rat and the mouse. Na_X_ immunostaining did not colocalized with the tanycytes of the funiculus separans zone or with GFAP-positive cells observed in the inverted pyramidal area extending from the ventral zone of the AP to the central canal. Na_X_ immunostaining was restricted to cells present in the three zones of the AP, which are the central, the lateral, and the junctional zone of the AP. The absence of vimentin and GFAP-positive cells in these zones strongly suggests that Na_X_ is expressed by neurons in the AP. The observation of Na_X_ immunostaining in the AP indicates that Na^+^ sensing intrinsically occurs in the medullary CVO, which supports the role of the AP in the central network of sodium sensing. The present data indicate that AP neurons not only relay the chemosensitive information regarding plasma sodium levels from the hepatoportal osmoreceptors but also likely serve as genuine sodium sensors similar to those found in the lamina terminalis. Hence, these neurons could directly forward sensory information to higher integrators such as the aldosterone-sensitive neurons in the nucleus tractus solitarius (NTS) or the FoxP2-immunoreactive neurons in specific divisions of the parabrachial nucleus (PBN; Geerling et al., 2006, 2011; Geerling and Loewy, 2007; Stein and Loewy, 2010). Moreover, the presence of Na_X_-expressing neurons in the AP may partly account for the neural activity observed in the rat AP after sodium ingestion but not after sodium depletion (Geerling and Loewy, 2007). Indeed, high plasma sodium levels will depolarize these sodium sensing neurons, which would likely lead to the observed Fos immunostaining in the AP. By contrast, sodium levels below the set point would hyperpolarize these neurons, thereby reducing the neural activity and the presence of Fos in the CVO.

### Na_X_ immunostaining in the SON and PVN

The presence of Na_X_ immunostaining in the magnocellular neurosecretory cells (MNCs) of the SON and PVN in the rat and mouse represents a major finding of this anatomical study. The expression of the Na_X_ channel by both the vasopressinergic and oxytocinergic MNCs was unexpected according to a previous mapping study of the Na_X_ gene in the mouse brain (Watanabe et al., 2000). However, this discrepancy may be related to the labeling method used in the two studies or to the abundance of the Na_X_-expressing cell population in the two rodent species. Watanabe et al. (2000) used a Na_X_ deficient mouse in which a *lacZ* reporter gene was inserted in frame into the mouse Na_X_ gene; accordingly, the sensitivity of this technique may not have been adequate for Na_X_ detection in smaller cell populations. Indeed, our data clearly indicate that the size of the Na_X_-immunopositive MNC population is higher in the rat than in the mouse. Intriguingly, *in situ* hybridization carried out with a Na_X_ riboprobe failed to reveal the presence of Na_X_ mRNA in the rat SON (Grob et al., 2004). However, RT-PCR experiments performed on micropunched tissues from both the SON and PVN in the present study showed a clear Na_X_-positive signal in the two nuclei. Similar differences in the level of Na_X_ expression were reported in the human hippocampus where the presence of Na_X_ mRNA was revealed by RT-PCR and not by *in situ* hybridization (Gorter et al., 2010). Taken together, our results indicate that basal level of Na_X_ mRNA in the MNC is weak, thereby suggesting a low turn-over of the protein in these neuroendocrine cells. Functionally, Na_X_ does appear to participate in the process of Na^+^ detection in the SON and PVN. It was previously shown that the MNCs were permeable to the Na^+^ ion, which was demonstrated with a N-terminal variant of the TRPV1 (transient receptor vanilloid 1; Voisin et al., 1999; Naeini et al., 2006). These electrophysiological recordings demonstrated that an increased extracellular Na^+^ concentration triggered an inward current with an amplitude that was strongly reduced (∼88%), but not abolished, by the application of gadolinium, which is a specific TRPV blocker (Voisin et al., 1999). The residual current was quantitated to be about 5 pA and might represent sodium influx through the Na_X_ channel. Indeed, such a current amplitude is well in the range of the sodium inward current flowing through Na_X_ in the MnPO neurons recorded *in situ* (Grob et al., 2004).

## Conclusion

The present study reveals the common and strategic Na_X_ expression in all the brain areas involved in the monitoring of plasma and CSF Na^+^ concentration in the rat and mouse. Beside the expected location of Na_X_ in the forebrain CVOs, Na_X_ was found in the AP, the ME and in the magnocellular neuroendocrine cells, which synthesize and release vasopressin and oxytocin to regulate systemic water and Na^+^ balance. The noticeable discrepancy concerns the Na_X_-expressing cell types in the network controlling Na^+^ sensing in the rat and mouse. The pattern of Na_X_ expression was clearly different in the nuclei of the lamina terminalis, because Na_X_ was expressed in neurons, ependymocytes, and tanycytes of the rat, whereas Na_X_ was restricted to ependymocytes and tanycytes in the mouse. In addition, Na_X_ was not expressed in the mouse MnPO. The difference in Na_X_ expression pattern and in Na_X_-expressing cell types strongly suggests that the mechanisms involved in systemic and central Na^+^ sensing are specific to each rodent species.

## Conflict of Interest Statement

The authors declare that the research was conducted in the absence of any commercial or financial relationships that could be construed as a potential conflict of interest.
